# Cerebrospinal Fluid Pressure Decreases with Older Age

**DOI:** 10.1371/journal.pone.0052664

**Published:** 2012-12-26

**Authors:** David Fleischman, John P. Berdahl, Jana Zaydlarova, Sandra Stinnett, Michael P. Fautsch, R. Rand Allingham

**Affiliations:** 1 Department of Ophthalmology, University of North Carolina, Chapel Hill, North Carolina, United States of America; 2 Vance Thompson Vision, University of South Dakota Medical Center, Sioux Falls, South Dakota, United States of America; 3 Department of Ophthalmology, Mayo Clinic, Rochester, Minnesota, United States of America; 4 Duke University Eye Center, Durham, North Carolina, United States of America; Zhongshan Ophthalmic Center, China

## Abstract

**Purpose:**

Clinical studies implicate low cerebrospinal fluid pressure (CSFP) or a high translaminar pressure difference in the pathogenesis of primary open angle glaucoma (POAG) and normal tension glaucoma (NTG). This study was performed to examine the effect of age, sex, race and body mass index (BMI) on CSFP.

**Methods:**

Electronic medical records from all patients who had a lumbar puncture (LP) performed at the Mayo Clinic from 1996–2009 were reviewed. Information including age, sex, race, height and weight, ocular and medical diagnoses, intraocular pressure (IOP) and LP opening pressure was obtained. Patients using medications or with medical diagnoses known to affect CSFP, and those who underwent neurosurgical procedures or where more than one LP was performed were excluded from analysis.

**Results:**

Electronic medical records of 33,922 patients with a history of having an LP during a 13-year period (1996–2009) were extracted. Of these, 12,118 patients met all entry criteria. Relative to mean CSFP at age group 20–49 (mean 11.5±2.8 mmHg), mean CSFP declined steadily after age 50, with percent reduction of 2.5% for the 50–54 age group (mean 11.2±2.7 mmHg, p<0.002) to 26.9% for the 90–95 group (mean 8.4±2.4 mmHg, p<0.001). Females had lower CSFP than males throughout all age groups. BMI was positively and independently associated with CSFP within all age groups.

**Conclusion:**

There is a sustained and significant reduction of CSFP with age that begins in the 6^th^ decade. CSFP is consistently lower in females. BMI is positively and independently associated with CSFP in all age groups. The age where CSFP begins to decline coincides with the age where the prevalence of POAG increases. These data support the hypothesis that reduced CSFP may be a risk factor for POAG and may provide an explanation for the mechanism that underlies the age-related increase in the prevalence of POAG and NTG.

## Introduction

Primary open angle glaucoma (POAG) is the most common form of glaucoma, a family of disorders with varying biological and environmental risk factors. Well-described risk factors for POAG include elevated intraocular pressure (IOP), advancing age, African and Hispanic ancestry [1], reduced central corneal thickness [1]–[5], and a positive family history. Recent studies suggest that lower cerebrospinal fluid pressure (CSFP) may contribute to the risk of developing open angle glaucoma, including both POAG and normal tension glaucoma [6]–[8]. The lamina cribrosa located at the anterior face of the optic nerve is situated between two pressurized compartments, the intraocular and subarachnoid spaces, and is the site of retinal ganglion cell axon loss in glaucoma. Both elevated IOP and reduced CSFP increase translaminar pressure. It is hypothesized that lower cerebrospinal fluid pressure acts similarly to elevated IOP at the optic nerve head, increasing risk for glaucomatous neuropathy. Retrospective and prospective clinical studies have found that CSFP is lower in patients with POAG and increased in patients with ocular hypertension (OHT) [6]–[9]. Importantly, this hypothesis offers an explanation for the mechanism of disease in patients with normal tension glaucoma (NTG), which occurs in the absence of statistically elevated IOP. Conversely, as elevated CSFP would theoretically reduce the translaminar pressure difference, higher CSFP could function as a protective factor for glaucoma in patients with OHT.

Even though the cerebrospinal fluid (CSF) has been studied for many decades, there is remarkably little published about what constitutes a “normal” CSFP. The primary method to determine CSFP in patients is to perform a lumbar puncture (LP). Since performing an LP carries some risk of adverse events to the patient, it has generally been reserved for patients suspected of harboring serious diseases and is rarely performed in otherwise healthy subjects. Consequently, current CSFP reference ranges have been generated from either small groups of volunteers or from larger, less well-characterized groups [10]–[12].

The purpose of the current study was to investigate the effect of age, sex, race, and body mass index on CSFP in a large dataset based on a long-standing electronic medical record system.

## Methods

### Patient Selection

This was a retrospective chart analysis from the Mayo Clinic's electronic medical records system of patients over age 20 years who underwent a diagnostic lumbar puncture from 1996 to 2009. Following Institutional Review Board approval granted from the Mayo Clinic in Rochester, Minnesota, a list of patients from December 1, 1996 to December 31, 2009 was generated by searching for diagnostic lumbar puncture by CPT code 62270. All subjects were de-identified according to Mayo Clinic protocol.

### Lumbar Puncture

At the Mayo Clinic (Rochester, MN) trained teams perform lumbar punctures in most cases. These teams use a standardized method that is performed similarly for all patients. Using this approach, patients are placed in the lateral decubitus position and either the L3 to L4 or L4 to L5 interspace is identified and anesthetized. A 3.5-inch 20-g spinal needle with a 3-way stopcock is inserted into the subarachnoid space. A 550-mm manometer is attached to the stopcock and the column of CSF fluid is allowed to equilibrate. The patient is asked to remain still and not to speak. For some patients, a bedside lumbar puncture may be difficult due to body habitus or due to other anatomical issues. In those cases, the lumbar puncture was performed by Radiology under fluoroscopic guidance. The electronic database extraction does not indicate which method was used for LP. In either method, the meniscus of the CSF fluid is read and reported in millimeters of water. For this study we converted millimeters of water to millimeters of mercury in order to simplify comparison with IOP for data analysis and discussion (1 mmHg = 13.6 mm H_2_0).

### Extracted Information

Information abstracted from this search included time and date of lumbar puncture, as well as the time and date of the CSF analysis. Patient's age, sex, race, ethnicity, height, and weight were obtained at the time of LP. A list of up to 15 separate current and past diagnoses was obtained as well as all medications. Blood pressures measured at the various visits of the patients who underwent LP were available for review. Only blood pressures recorded within one month of the lumbar puncture (either before or after) were included for analysis. The blood pressure measured closest to the time of lumbar puncture was selected. If blood pressure was measured one day before and one day following LP we used the earlier measurement for analysis.

All admissions or procedures performed at the Mayo Clinic were reviewed for each patient. BMI for each patient was calculated manually [BMI = (weight in kilograms)/(height in meters)^2^] and rounded to the nearest whole number. BMI less than 10 and greater than 50 were excluded as these values were felt to be the result of data entry errors. The height and weight used for calculation of BMI was performed from measurements recorded in the hospital stay associated with the LP.

### IOP Data

Patients who met initial screening criteria and had an ophthalmologic exam including IOP measurements within one-year of the LP were identified. The method of measurement of IOP was not recorded in the dataset. Patients with POAG or other eye disease were not excluded from this study (with the exception of idiopathic intracranial hypertension since it is associated with altered CSFP). If repeat ophthalmologic exams were performed, the IOP measurement closest to LP data was used for evaluation. Right eye, left eye, and the average of both eyes IOP were regressed against age.

### Screening

Information from specific patients was given a unique, randomly generated identifier and entered into a database. Patients with medical conditions, head trauma, taking medications known to alter CSFP, or having a neurosurgical procedure were excluded. A total of 134 different medications and diagnostic codes of medical conditions that might affect CSFP were used for manual screening of all electronic medical records. Filters were applied regardless of time sequence leading to the LP. For example, if a patient developed a condition known to alter CSFP at any time point, they were excluded from the study. All patients with multiple LPs were excluded. Patients with CSFP values <60 or >250 mmH_2_O (<4.41 mmHg or >18.38 mmHg), considered outside the accepted normal range, were excluded from analysis [13].

### Analysis

Subjects who met all inclusion criteria were stratified into 1, 5 and 10-year age groups. For the purpose of this study, we used World Health Organization criterion for the age of adulthood, which is ≥20 years [14]. Mean CSFP and standard deviation was calculated for each age group. A two-tailed student's t-test was used to measure the difference between each group and to determine significance. A p-value<0.05 was used for statistical significance.

Multivariate linear regression models, both segmented and non-segmented by age, were created for multiple sets of variables. Age, BMI, sex, race (Black, Caucasian, Asian, Other), presence of diabetes mellitus (DM), mean arterial pressure (MAP), and time of LP were regressed for the purpose of determining independent association with CSFP.

## Results

The CPT database extraction protocol identified electronic medical records for 33,922 patients who had diagnostic lumbar punctures performed between December 1, 1996 and December 31, 2009. The number meeting initial inclusion criteria was 13,715, which is 40.4% of the original extraction group. The final dataset used for analysis contained 12,118 patients after exclusion for CSFP values out of the normal range, age less than 20, and BMI <10 and >50. The distribution and demographic data of the analysis dataset are listed in **Table 1** **and** **2**. The age distribution of the study group was evenly distributed with mean age of 54.4 years±15.2, with a median age of 55. Caucasians were the largest group in this population (79.1%), followed by “Unknown” (17.1%), Black (1.4%), and “Other” (1.0%). Regarding ethnicity, 0.6% self-identified as Hispanic, 17.4% as Not-Hispanic, and 81.9% were documented as “Unknown.” Sex was evenly distributed between males and females, 49% and 51% respectively. While indications for LP were not included in the extracted data, the primary diagnosis was available for the medical visit where the LP was performed. There were a total of 1,404 unique diagnoses recorded among the patients included in the final dataset. Of all the patients included in the final dataset, over 69,041 diagnoses were listed. The top ten primary diagnoses for the analysis dataset are listed in **Table 3**.

**Table 1:** Derivation of the final study population after adoption of all entry criteria.

Disposition	N	Percent
Extracted in data base	13715	100
Excluded*	1597	11.6
- Out of range CSFP values (<4.4 or >18.4)	1152	8.4
- Age ≤19	532	3.9
- BMI ≤10 or ≥50	27	0.2
Included in overall study	12118	88.4
Included in analysis of BMI out of overall included in study	4314	35.6

* *114 were in two exclusion categories.*

**Table 2:** Demographics and disposition of study patients following final screening.

Demographic	Statistic	All Study Patients	Patients with Recorded BMI
	N	12118	4314
Age	Mean (SD)	54.4 (15.2)	55.0 (15.2)
	Min, Median, Max	20, 55, 95	20, 55, 91
BMI	N	4314	
	Mean (SD)	26.7 (5.2)	26.7 (5.2)
	Min, Median, Max	10.1, 26.1, 49.1	10.1, 26.1, 49.1
Gender	N (%)		
- Female		6224 (51)	2250 (52)
- Male		5894 (49)	2064 (48)
Race	N (%)		
- American Indian/Eskimo		44 (0.4)	20 (0.5)
- Black		171 (1.4)	73 (1.7)
- Caucasian		9586 (79.1)	3409 (79.0)
- Native Hawaiian/Pacific Islander		4 (0.03)	4 (0.1)
- Other		121 (1.00)	43 (1.0)
- Asian		81 (0.7)	23 (0.5)
- Unknown		2073 (17.1)	726 (17.9)
- Choose not to disclose		38 (0.3)	16 (0.4)
Ethnicity	N (%)		
- Hispanic		73 (0.6)	29 (0.7)
- Not Hispanic		2107 (17.4)	1036 (24.0)
- Unknown		9919 (81.8)	3238 (75.1)
- Choose not to disclose		19 (0.2)	11 (0.2)

**Table 3:** Top ten primary diagnoses at visit of lumbar puncture for patients ages 20–95.

	Primary Diagnosis	N	%
1	IDIOPATHIC PERIPHERAL NEUROPATHY, NOS	887	7.69%
2	SKIN SENSATION DISTURBANCES	424	3.67%
3	MULTIPLE SCLEROSIS	397	3.44%
4	HEADACHE	370	3.21%
5	AMYOTROPHIC SCLEROSIS	367	3.18%
6	ABNORMALITY OF GAIT	286	2.48%
7	SPINAL CORD DISEASE NOS	250	2.17%
8	OTHER MALAISE & FATIGUE	195	1.69%
9	PERSISTENT MENTATION DISORDER NEC	164	1.42%
10	HYPERTENSION NOS	161	1.40%

*N: Number of Patients; % = Percent of all primary diagnoses.*

**Figure 1:**
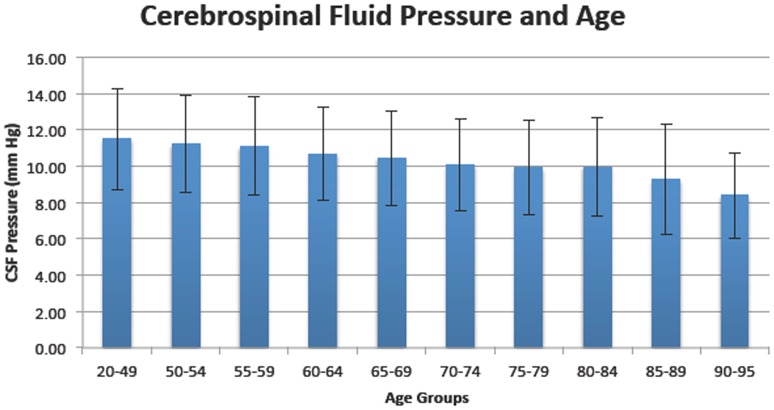
Mean CSFP within specific age groups. Bars represent one standard deviation.

There was no difference in CSFP values measured by 1, 5, or 10 year age groupings between ages 20 to 49. Therefore we used mean CSFP for the 20–49 age group as a baseline to compare to subsequent five-year age groups. Compared to the mean CSFP for age group 20–49, a statistically significant difference in mean CSFP was evident in the 50–54 age group (2.5%, p<0.002). (**Table 4** **and** **Figure 1**) Mean CSFP progressively decreased for each subsequent 5-year age group. CSFP reduction was 13.3% (1.53 mmHg) at age 80, 19.4% (2.23 mmHg) at age 85, and 26.9% (3.09 mmHg) at age 90–95. A similar analysis, which grouped the study population as young adults (20–49 years), late-middle age (50–69), and older adults (greater than 70 years), was performed. The 20–49 group had a mean CSFP of 11.5 mm Hg±2.7, which was 5.3% (10.9 mmHg ±2.7; p<0.0001) higher than the 50–69 group, and 13.1% (10.0 mm Hg ±2.6; p<0.0001) higher than the group of older adults, or those greater than 70 years.

**Table 4:** Mean cerebrospinal fluid pressure (mm Hg) by age categories.

CSF Pressure by Age Category				
Age Category	Statistic		P-Value*	Percent Lower**
20–49	N	4592		
	Mean (SD)	11.49 (2.75)		
	Min/Median/Max	4.41, 11.47, 18.38		
50–54	N	1402		
	Mean (SD)	11.20 (2.67)	0.001	2.5
	Min/Median/Max	4.41, 11.03, 18.38		
55–59	N	1435		
	Mean (SD)	11.09 (2.71)	<0.001	3.5
	Min/Median/Max	4.41, 11.03, 18.38		
60–64	N	1150		
	Mean (SD)	10.68 (2.60)	<0.001	7.0
	Min/Median/Max	4.41, 10.59, 18.38		
65–69	N	1269		
	Mean (SD)	10.46 (2.60)	<0.001	9.0
	Min/Median/Max	4.41, 10.29, 18.38		
70–74	N	1084		
	Mean (SD)	10.08 (2.51)	<0.001	12.3
	Min/Median/Max	4.41, 10, 18.38		
75–79	N	781		
	Mean (SD)	9.93 (2.62)	<0.001	13.6
	Min/Median/Max	4.41, 9.85, 18.24		
80–84	N	318		
	Mean (SD)	9.96 (2.72)	<0.001	13.3
	Min/Median/Max	4.56, 9.71, 18.38		
85–89	N	79		
	Mean (SD)	9.26 (3.05)	<0.001	19.4
	Min/Median/Max	4.71, 8.68, 18.38		
90–95	N	11		
	Mean (SD)	8.40 (2.35)	<0.001	26.9
	Min/Median/Max	5.29, 8.24, 13.24		

Percent difference is compared against the 20–49 year age group.

* *P-value based on F-test of difference in CSF means between each older age category and age category 20–49.*

** *Percentage lower of mean in age category than mean in 20–49 age category.*

To determine the relationship of age and BMI on CSFP, a two-way analysis of variance was fit to the data. For the subset of subjects with recorded BMI, stratifications were created by evenly distributing the *N* of the subgroups. Both age (p<0.001) and BMI (<0.001) were significant predictors of CSFP. There was no difference in the trend of CSFP in the BMI categories over the age categories (p = 0.611): overall, CSFP was negatively correlated with advancing age after age 50 and was positively correlated with BMI regardless of age and within all age groups. (**Table 5**) This finding was consistent using either segmented (correlation: 0.223, standard error [SE]: 0.01; p<0.001) or non-segmented (correlation: 0.23, SE: 0.01; p<0.001) linear regression models, both of which confirmed that BMI and age are independently associated with CSFP.

**Table 5:** Bivariate analysis of mean cerebrospinal fluid pressure (mm Hg) as a function of BMI within specific age groups.

Age	N	Mean CSFP	BMI	N	Mean CSFP
20–42	861	11.56	10.1–22.3	241	9.92
			22.3–24.8	167	11.01
			24.8–27.5	148	11.78
			27.5–30.8	148	12.59
			30.8–49.1	157	13.49
42–51	767	11.31	10.1–22.3	156	9.37
			22.3–24.8	154	10.76
			24.8–27.5	143	11.16
			27.5–30.8	127	12.28
			30.8–49.1	187	12.82
51–60	953	11.04	10.1–22.3	154	9.09
			22.3–24.8	197	10.00
			24.8–27.5	170	10.74
			27.5–30.8	200	11.78
			30.8–49.1	232	12.81
60–69	796	10.61	10.1–22.3	139	8.60
			22.3–24.8	145	9.60
			24.8–27.5	172	10.52
			27.5–30.8	181	11.26
			30.8–49.1	159	12.63
69–91	937	9.84	10.1–22.3	169	8.18
			22.3–24.8	202	9.19
			24.8–27.5	227	10.07
			27.5–30.8	210	10.50
			30.8–49.1	129	11.57

**Figure 2:**
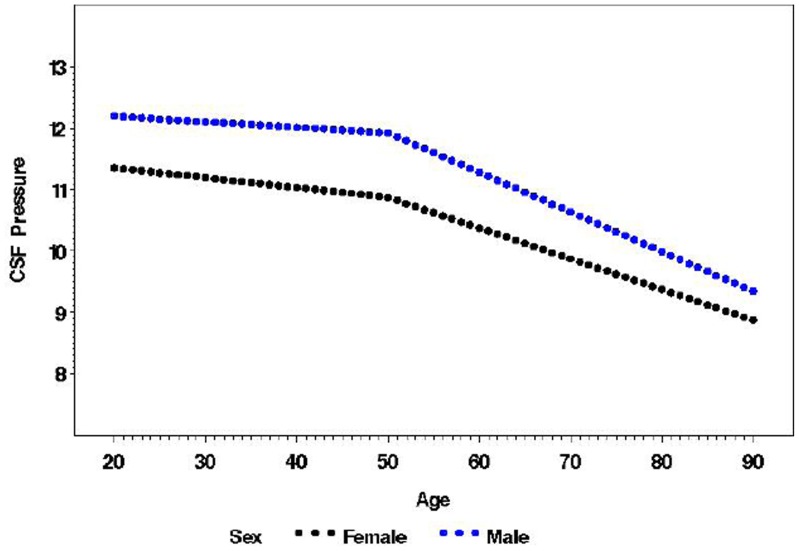
Segmented multivariate linear regression model CSFP (mm Hg) by age and sex. For each year of increase in age, CSFP decreased 0.009 mmHg (p = 0.132) for age <50 and by 0.056 mmHg, (p<0.001) for age greater than 50. Prior to age 50, the slope is not different between males and females (p = 0.369). After age 50, the gender difference in the slope is borderline significant (p = 0.067). The r^2^ of this model is 0.071.

CSFP was significantly different between sexes. On average, males have a higher CSFP than females by approximately 0.9 mmHg. The rate of change of CSFP with age for males and females was the same (ages 20–50, p = 0.369; ages 50–95, p = 0.067). Although this difference was significant, sex as a variable explains only a small portion of the variability of CSFP in this dataset (r^2^ = 0.07, p<0.0001). (**Figure 2**)

We attempted to look at the effect of race on CSFP with age. Overall, mean CSFP was higher in Black patients compared to Caucasians (12.01 mmHg ±2.9 and 10.9 mmHg ±2.7, respectively). The N for all Black patients within this study was 171, or 1.4% of the dataset. The N was further reduced by incorporation of other independent factors such as BMI for regression analyses. The lack of statistical power precluded our ability to determine the role, if any, between race and ethnicity with CSFP.

Other variables such as blood pressure, diabetes, and the time of lumbar puncture were examined. Mean arterial pressure (MAP) within one month of LP was positively, but weakly, correlated with CSFP (correlation: 0.016, SE: 0.00; p<0.0001). A statistically significant, but weakly positive correlation exists between MAP and age in this study group. (r^2^ = 0.02, p<0.0001). Blood pressure data was available for approximately one third of patients (N = 4197 or 34.6%), and the respective model explains only a small amount of the observed variability in CSFP. CSFP was not correlated with time of lumbar puncture (correlation: 0.012, SE: 0.01; p = 0.3881) or diabetes mellitus status (correlation: −0.213, SE: 0.15; p = 0.149).

**Figure 3:**
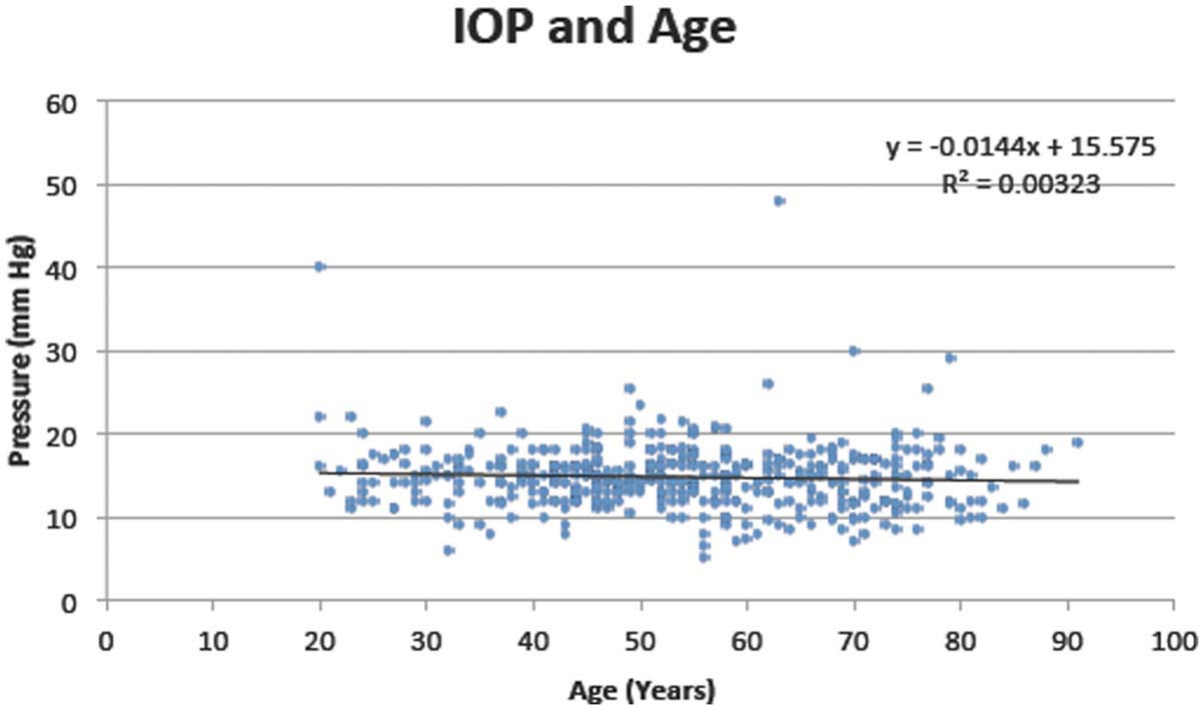
Average intraocular pressure against age. Formula for the regression: y = −0.0144×+15.575, r^2^ = 0.003.

A subgroup of patients (N = 441) who had IOP and CSFP recorded were selected for IOP and CSFP analysis. Whereas CSFP changes significantly with age, there was no statistically significant difference in mean IOP with age in this group. (**Table 6**, **Figure 3**).

**Table 6:** Intraocular pressure, cerebrospinal fluid pressure, and translaminar pressure difference by age groups.

Age	IOP AVG	CSFP	N	TLPd	Difference*	P value
**20–49**	14.9	10.5	178	4.4		
**50–59**	14.9	11.3	106	3.6	0.41%	0.884
**60–69**	14.7	9.9	75	4.8	1.39%	0.746
**70–79**	14.7	8.3	65	6.4	1.43%	0.726
**>80**	13.8	7.6	17	6.2	7.36%	0.193

The percentage difference is calculated by mean IOP average of each age group against mean IOP average at age 20–49; significance measured by two-tailed Student's t-test. *IOP: intraocular pressure; CSFP: cerebrospinal fluid pressure; TLPd: translaminar pressure difference*.

## Discussion

There is a growing body of evidence that indicates CSFP is lower in patients with primary open angle glaucoma compared to non-glaucomatous subjects and additionally, is lower in normal tension versus the high tension form of POAG.^6–8^ If CSFP is related to the pathogenesis of POAG then it is important to explore factors that affect CSFP. In this study we report the effect of age on CSFP. We have found that mean CSFP is stable for the first 50 years of life after which there is a steady decline. The observed change in CSFP is substantial, with the mean reduction in measured CSFP being 3.1 mm Hg (26.9%) by age 90. We also determined that the effect of age on CSFP is independent of BMI, and that BMI is positively and independently associated with CSFP.

The few studies that have examined CSFP and age failed to find a relationship [11], [12], [15], [16]. In a study of 200 mostly syphilitic subjects, performed by Masserman in 1935, no significant correlation was found between age and resting cerebrospinal fluid pressure in those aged 16 to 48 [7]. Our study corroborates CSFP stability in this age group. Studies by Ekstedt and, more recently, Malm were conducted on 100 and 40 subjects respectively [11], [16]. No correlation was found between CSFP and age in these studies. However, after stratification by age, it is likely that these studies were underpowered to observe this phenomenon. In contrast, the current study was based on measurements obtained on 12,118 subjects, a dataset at least two orders of magnitude larger than previous reports.

Prospective and retrospective studies have shown that BMI is positively associated with CSFP in different populations [17]–[19]. In a clinical interventional study of 71 patients in China, Ren and colleagues identified a positive correlation between BMI and CSFP [18]. These investigators did not find a significant relationship between lower CSFP with age. In the present study, we found that BMI and age independently influence CSFP. However, whereas CSFP declines beginning in the 6^th^ decade of life, BMI is positively associated with CSFP in every age group we studied. A biomechanical explanation for the relationship between cerebrospinal fluid pressure and body mass index has been suggested for patients with idiopathic intracranial hypertension (IIH). Studies of IIH suggest that obesity, in particular central obesity, increases intra-abdominal pressure, which ultimately causes an increase in venous pressure and consequently intracranial pressure [20], [21]. It is plausible that a similar mechanism is in play in the entire spectrum of BMI. Interestingly, it has also been reported that lower BMI is a possible risk factor for glaucoma, or conversely, increased BMI may be protective [22]–[24].

We have found that CSFP is higher in males compared to females within all age groups after controlling for BMI and age. Although males consistently have higher CSFP values, the model was unable to pronounce the relationship between advancing age and sex. If lower CSFP is a risk factor for POAG, this would suggest that women are at increased risk for POAG. However most population-based studies have found little difference in prevalence and incidence between sexes [25]–[27]. In fact, Rudnicka and colleagues' meta-analysis of population-based studies concluded that, in general, men have a higher prevalence of POAG than women (OR = 1.37 [1.22, 1.53]) [28], [29]. On the other hand, Drance reported that female patients with normal tension glaucoma have a more rapid progression than males [30]. It may be that the effect of sex on CSFP, 6.5% lower mean CSFP in women in this study, is too small in existing populations-based studies. It is also possible that other variables, such as hormonal influences, exert an effect on POAG risk [23].

We examined the effect of race and CSFP. Mean CSFP appeared to be higher in Black patients compared to Caucasians. However, the number of Black patients in this dataset is small (1.4% of the study population) and is consequently inadequate to draw a meaningful conclusion on this important topic. For similar reasons, the effect of the time lumbar puncture is performed, mean arterial pressure, and diabetes mellitus on CSFP are not possible. Well-powered studies of this type, with a particular focus on the effect of race would be of great interest.

We have found that CSFP progressively decreases after age 50. This suggests that either the resistance to CSFP outflow is reduced or that CSF production decreases with age. Cerebrospinal fluid is produced in the choroid plexus within the third, fourth, and inferior horn of the lateral ventricles, and drains in the arachnoid villi into the cerebral venous system, as well as through lymphatic channels [31]–[35]. There is no reported evidence that CSF outflow resistance decreases with age, rather most studies report CSF flow resistance increases [34], [36]. However, there is evidence that the choroid plexus undergoes aging changes leading to decreased CSF production [37]–[39].

Levels of vasopressin, a hormone that regulates the choroid plexus, is affected by aging [40]. Vasopressin receptor (V1) activation in the choroid plexus has been shown to decrease blood flow and reduce CSF secretion. Vasopressin-secreting neurons show increased activity with aging. Vasopressin levels are elevated in the CSF of old rats and in plasma of older humans [41]. May and colleagues examined the rate of CSF production in 7 young and 7 elderly healthy normal volunteers [37]. Although opening CSF pressures were similar between the two groups, the mean CSF production was reduced by over 50% in the older age group (0.19±0.07 versus 0.41±0.24 ml/min, p<0.02). These data support the role of reduced CSF production rather than increased drainage as the most likely cause of lower CSFP with age.

We hypothesize that the decline in CSFP with age in conjunction with essentially stable IOP would produce an age-related increase in the translaminar pressure gradient. These findings carry potentially major implications for risk of POAG with age. A study by Morgan et al found that the mechanical effect of altering CSFP on the lamina cribrosa is equivalent to or greater than altering IOP [42], [43]. Assuming equivalence for IOP and CSFP on the translaminar pressure gradient, we can examine the effect of a similar rise in IOP on POAG risk using data provided by population-based studies. In the Baltimore Eye Survey the relative risk of POAG increased from 2.8 for an IOP group of 19–21 mmHg to 12.8 for IOPs of 22–24 mmHg, a 4 fold increase in risk produced by an average 3 mmHg increase in IOP [44]. The Beaver Dam Eye Study reported that patients with glaucoma had an average IOP of 20.1 mmHg compared to 15.3 mmHg in unaffected controls, which represents a mean difference of approximately 5 mmHg [45]. The Tajimi Eye Study [46] is of particular interest since over 90% of untreated POAG cases had an IOP at diagnosis within the normal range. These investigators found that a rise of 4 mmHg increased POAG risk 2 fold even within the normal IOP range. These studies support the notion that relatively small increases in IOP and therefore the translaminar pressure gradient, similar to those induced by age on CSFP observed in this study, may be associated with substantial changes in relative risk for POAG.

The time frame for age-dependent reduction in CSFP mirrors that seen for the rise in POAG prevalence in population-based studies performed in the United States and globally. In the United States, the Baltimore Eye Survey, Beaver Dam Eye Study, and the Los Angeles Latino Eye Study have all found that POAG prevalence begins to rise in the 5^th^ and 6^th^ decades and progressively increases through the 8^th^ or 9^th^ decades [1], [44], [45], [47]–[49]. Studies conducted in Australia, Europe, China, and Japan describe similar trends [50]–[59]. Taken together the hypothesized effect of a reduction of CSFP with age is compatible with observed increases in the prevalence of POAG that are consistently observed in populations throughout the world. This hypothesis may be particularly applicable in the Hispanic and Japanese populations where the great majority, 80% and 92%, respectively, of those with POAG have a normal IOP at the time of diagnosis. Interestingly, a genome-wide association study of POAG identified a genetic locus on chromosome 8q22 that is strongly associated with normal pressure glaucoma and contains putative regulatory sites that are active in choroid plexus epithelial cells, the cell type responsible for CSF production [60].

We recognize several limitations in this study. Although the dataset is robust in size this is still subject to the challenges encompassed by a retrospective analysis. While diagnoses were available for each of these patients, it was not possible to review the actual individual medical records for accuracy, although studies of accuracy between ICD-9 codes and actual medical records have demonstrated acceptable congruency [61], [62]. Some information from the CSF analysis, including cell counts and differentials, were not available so it is possible that subjects with abnormal CSF parameters were inadvertently included in the analysis. Similarly, although data were carefully reviewed to exclude conditions that affect CSFP, it is possible that some subjects developed conditions for which they would have been excluded at a later date or some conditions may exert a currently unknown influence on CSFP.

Another important consideration is the interpretation of the values reported in this study. Lenfeldt found that CSFP measured by lumbar puncture accurately represents intracranial pressure as long as the patient is in the lateral decubitus position [63]. However, the intracranial pressure differs from that within the orbital CSF space due to differences in fluid dynamics. Furthermore, altering position, for example upright to supine posture, induces substantial changes in CSFP [64], [65]. These positional CSFP changes occur routinely, as do changes from diurnal variation.

This study utilized a “snapshot” in time for a single individual so these values do not reflect CSFP as a function of time for any particular patient or group of patients. Additionally, this study did not discriminate between bedside lumbar punctures in the lateral decubitus position and fluoroscopy-guided lumbar punctures which may account for minor differences in pressures. Despite these limitations, we feel that the robust patient dataset compensates for many of these limitations.

Finally, the coefficient of determination (r^2^) in the majority of the regression models indicates that the group of predictors chosen (age, sex, BMI, race) together are only modestly predictive of CSFP. This highlights the need to investigate and identify other variables that may impact CSFP.

In conclusion, these data support the hypothesis that CSFP may play a role in the pathogenesis of POAG through its contribution to translaminar pressure. CSFP decreases significantly and steadily after age 50. The observed change in CSFP with age parallels the rise in prevalence of POAG and is of a magnitude that is sufficient to produce a significant increased risk for glaucoma. This finding offers a previously unrecognized mechanism to help explain age-related risk for POAG in general, and for the large number of patients with statistically normal IOP in particular. If cerebrospinal fluid pressure is in fact an important factor in the pathogenesis of glaucoma, this could possibly introduce new avenues for therapeutics, assessment of glaucoma risk and estimation of progression.
